# BCCT: A GUI Toolkit for Brain Structural Covariance Connectivity Analysis on MATLAB

**DOI:** 10.3389/fnhum.2021.641961

**Published:** 2021-04-20

**Authors:** Qiang Xu, Qirui Zhang, Gaoping Liu, Xi-jian Dai, Xinyu Xie, Jingru Hao, Qianqian Yu, Ruoting Liu, Zixuan Zhang, Yulu Ye, Rongfeng Qi, Long Jiang Zhang, Zhiqiang Zhang, Guangming Lu

**Affiliations:** ^1^College of Automation Engineering, Nanjing University of Aeronautics and Astronautics, Nanjing, China; ^2^Department of Radiology, School of Medicine, Jinling Hospital, Nanjing University, Nanjing, China; ^3^Sleep Assessment Unit, Department of Psychiatry, Faculty of Medicine, Chinese University of Hong Kong, Hong Kong, China; ^4^State Key Laboratory of Analytical Chemistry of Life Science, Nanjing University, Nanjing, China

**Keywords:** structural covariance connectivity, causal network analysis of structural covariance, modulation, winner-take-all, GUI

## Abstract

Brain structural covariance network (SCN) can delineate the brain synchronized alterations in a long-range time period. It has been used in the research of cognition or neuropsychiatric disorders. Recently, causal analysis of structural covariance network (CaSCN), winner-take-all and cortex–subcortex covariance network (WTA-CSSCN), and modulation analysis of structural covariance network (MOD-SCN) have expended the technology breadth of SCN. However, the lack of user-friendly software limited the further application of SCN for the research. In this work, we developed the graphical user interface (GUI) toolkit of brain structural covariance connectivity based on MATLAB platform. The software contained the analysis of SCN, CaSCN, MOD-SCN, and WTA-CSSCN. Also, the group comparison and result-showing modules were included in the software. Furthermore, a simple showing of demo dataset was presented in the work. We hope that the toolkit could help the researchers, especially clinical researchers, to do the brain covariance connectivity analysis in further work more easily.

## Introduction

The brain is a connectome that collects network architectures by fragmental and coalescent organizations (Bullmore and Sporns, 2009). The human brain could be described by anatomical pathways and functional interactions among distinct brain regions (Bullmore and Sporns, 2009; Sporns, 2011). The human brain connectome can be separated into functional connectivity based on signal process (Biswal et al., 1995; Liao et al., 2010; Sporns, 2018), structural connectivity based on fiber tracts (Liao et al., 2011; Zhang et al., 2011a; Watson et al., 2019), and covariance connectivity based on structural covariance analysis (He et al., 2007; Zhang et al., 2011b; Liao et al., 2013). The covariance connectivity, mainly referred to as the structural covariance network (SCN) constructed by morphological images, could be used to measure the synchronized topological patterns of brain regions in a long-range time period (He et al., 2007; Liao et al., 2013; Zhang et al., 2017; Xu et al., 2020). Using a cross-sectional morphological image dataset, the structural covariance connectivity could measure the undergoing pathological processes between brain regions during development, plasticity, or diseases (He et al., 2007; Seeley et al., 2009; Sanabria-Diaz et al., 2010; Liao et al., 2013; Jiang et al., 2018; Li et al., 2019).

Recently, new techniques have expended the breadth of SCN. Based on the Granger causality algorithm, the causal analysis of structural covariance network (CaSCN) could be used to detect the progression patterns of SCN (Zhang et al., 2017; Jiang et al., 2018; Li et al., 2019; Guo et al., 2020). The strategy of CaSCN was to assign the temporal order to the structural covariance analysis (Zhang et al., 2017). The temporal order could be based on the variables with sequential order, such as the duration of diseases or disorders, age of participants, etc., (Zhang et al., 2017). With the temporal order, the Granger causality analysis (GCA) could be used to calculate the influences among the brain regions (Zhang et al., 2017). The winner-take-all and cortex–subcortex covariance network (WTA-CSSCN) was the combination of winner-take-all strategy and structural covariance analysis between cortex and subcortex regions (Xu et al., 2020). It could be used to build the parcelations of subcortex regions according to the cortex parcelations. And it could provide novel insight of the subcortex by using structural covariance analysis (Xu et al., 2020). Additionally, modulation analysis of structural covariance network (MOD-SCN) was developed for the detection of the influence of clinical variables onto the structural covariance connectivity (Bernhardt et al., 2014; Sharda et al., 2016; Valk et al., 2017; Xu et al., 2020). These developments of SCN widely extended the applications of the technique.

However, until now, there was no dedicated software for the analysis of brain structural covariance connectivity. Meanwhile, it was difficult to do the statistical analysis of SCN, especially for the clinical researchers. Hence, we developed this user-friendly graphical user interface (GUI) software, Brain Covariance Connectivity Toolkit (BCCT), which was based on MATLAB platform, to operate the related process of structural covariance connectivity. We hope it could help researchers who would like to do the structural covariance analysis in their studies.

## Interface and Main Functions of the Brain Covariance Connectivity Toolkit

Brain Covariance Connectivity Toolkit was developed by the Department of Radiology, Jinling Hospital, Medical School of Nanjing University. It was scripted on the MATLAB platform. It is suggested that the MATLAB version is later than R2014a. It could be downloaded from the website of github^1^. The supported operation systems include the Linux and Windows systems, while the MAC system has not been tested. It is suggested that the memory (RAM) should be larger than 8 GB for running the toolkit for seed-to-brain SCN analysis. It would be better that the RAM is larger than 16 GB for the large voxel and vertex numbers of gray matter volume (GMV) in MNI space and thickness in fsaverage space. It would cost large RAM and time for the permutation test. The toolkit needs spm^2^, freesurfer matlab tools^3^, and SurfStat toolkit^4^ for the I/O operation of the related image dataset.

Four kinds of SCN methods were included in the toolkit: SCN, CaSCN, MOD-SCN, and WTA-CSSCN. Apart from the WTA-CSSCN, the other SCN methods could deal with the morphological image datasets either on volume space or on surface space. The WTA-CSSCN could only process the volume-based morphological dataset currently.

**FIGURE 1:**
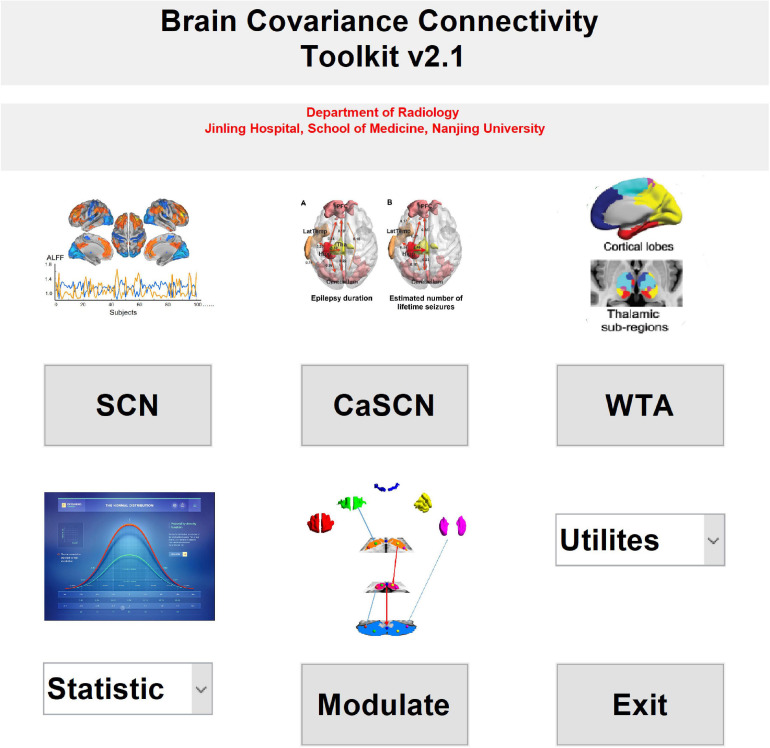
The main interface of the brain covariance connectivity toolkit (BCCT) toolkit. CaSCN, causal analysis of structural covariance network; SCN, structural covariance network; WTA, winner-take-all.

Meanwhile, two kinds of statistical analysis methods were included in the toolkit, the interaction analysis (Lerch et al., 2006; Valk et al., 2017; Xu et al., 2020), and the permutation test (Liao et al., 2013; Teipel et al., 2016; Xu et al., 2020). Currently, the software only supported the comparison between two groups. Additionally, the simple result-showing mode was included in the utilities of the toolkit (Figure 1). Also, the utilities included the mask generation function and help documents.

For SCN, CaSCN, and MOD-SCN, the input datasets should be the processed morphological indices, such as GMV from voxel-based morphometry (VBM) analysis^5^ /Computational Anatomy Toolbox toolkit (CAT12)^6^, brain area, thickness from FreeSurfer software (see text footnote 3), etc. The “ROI signals” were conducted with the mean values of region of interest (ROI) of the morphological indices in participants, and the “voxel/vertex signal” was produced by the value of the related vortex/vertex of the indices in participants.

There were four kinds of ROI definitions in the toolkit: the MNI coordinate mode, the ROI image mode, the mat mode, and the ASCII text mode.

After extracting the “ROI signals” and/or whole brain “voxel/vertex signal,” the related analysis methods of different SCN modes were used for further analysis: correlation analysis for SCN, GCA for CaSCN, and generalized linear model (GLM) analysis for MOD-SCN.

**FIGURE 2:**
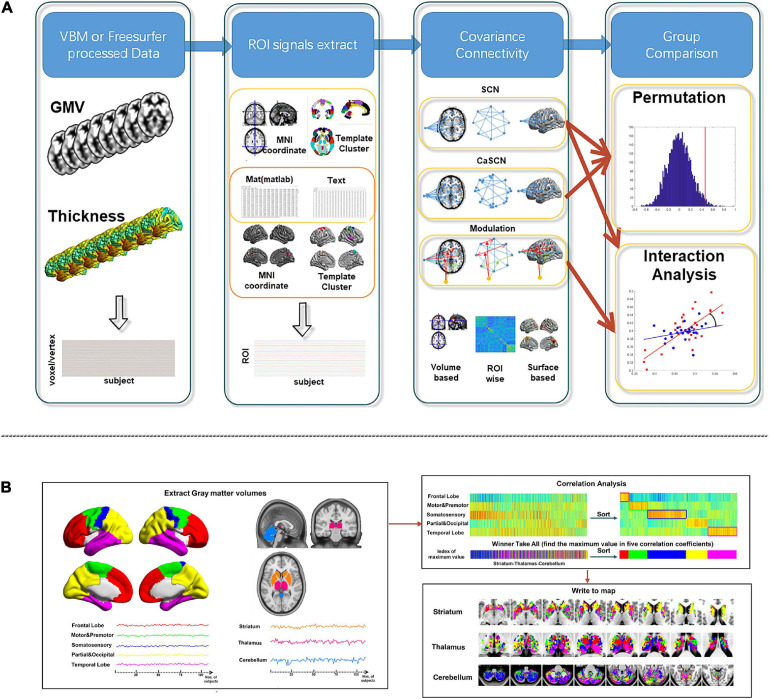
Workflow of BCCT toolkit. **(A)** Workflow of SCN, CaSCN, and modulation analysis of structural covariance network (MOD-SCN). **(B)** Workflow of winner-take-all and cortex–subcortex covariance network (WTA-CSSCN). Here, the gray matter volume (GMV) from VBM/CAT12 could be used for the voxel-wise seed-to-brain analysis of volume-based morphological images and region of interest (ROI)-wise analysis of volume-based morphological images. The thickness from FreeSurfer could be used for the vertex-wise seed-to-brain analysis of surface-based morphological images.

Then, the statistical analysis was applied to the group comparison analysis. For SCN and MOD-SCN, the interaction analysis could be used for the comparison. And for SCN and CaSCN, the permutation test could be used for the comparison (Figure 2A).

For WTA-CSSCN, the “ROI signals” were extracted from the parcelation of the cortex. Then, the correlation analysis was used to gain the correlation coefficients between “ROI signals” of the cortex and the “voxel signals” of the subcortex structure. Each voxel of the subcortex structure was labeled according to the cortical parcelation with the highest correlation coefficient, respectively. Then, the permutation test was used for the group comparison of the connectivity numbers of the subcortex structure in WTA-CSSCN (Figure 2B).

## Structural Covariance Network

Structural covariance network was the basic function of the brain structural covariance analysis. The SCN in the toolkit was constructed by correlation analysis. Two kinds of SCN were implemented in the toolkit: the voxel-/vertex-wise seed-to-brain structural covariance connectivity of volume-/surface-based morphological images and ROI-wise structural covariance connectivity of volume-based morphological images. Additionally, the regression of variables of no interest could be selected in the analysis. According to the advisement of VBM/CAT, the total intracranial volume (TIV) should be regressed out during the morphological analysis. There were two kinds of correlation methods in the toolkit, the Pearson correlation and partial correlation analysis. It was noted that it should make sure that the number of participants was larger than the number of the ROIs.

## Causal Analysis of Structural Covariance Network

Causal analysis of structural covariance network was firstly developed in 2017 (Zhang et al., 2017). In the study, the duration of disease was selected as the sequential order of participants. The GCA was applied to the seed-to-brain analysis and ROI-wise analysis. There were two kinds of GCA algorithms in the toolkit, one was the residual-based GCA (John, 1982; Goebel et al., 2003; Wu et al., 2013) and the other was the coefficient-based GCA (Chen et al., 2009; Zang et al., 2012).

The formulas of the residual-based GCA are as follows:

$$Y_{n}=\beta \,Y_{n-k}+\varepsilon$$

$$Y_{n}=\alpha \,X_{n-k}+\bar{\beta }\,Y_{n-k}+\delta$$

Here, *Y*_*n*_ represents the current value of *Y* signal, *X*_*n–k*_ and *Y*_*n–k*_ represent the former values of *X* and *Y* signals with *k*-order, and ε and δ represent the residual of the linear fit modes. When ε > δ, the *X* shows Granger causal effect on *Y*. The residual-based GCA is defined like this:

$$F_{x\to y}=\frac{\ln\left(\varepsilon \right)}{\ln\left(\delta \right)}$$

The formula of coefficient-based GCA is as follows:

$$Y_{n}=\alpha \,X_{n-k}+\beta \,Y_{n-k}+\varepsilon$$

Here, *Y*_*n*_ represents the current value of *Y* signal, *X*_*n–k*_ and *Y*_*n–k*_ represent the former values of *X* signal and *Y* signals with *k*-order, and ε represents the residual of the linear fit modes. The coefficient of α represents the causal effect of *X* to *Y*.

According to the distributions of the residual-based GCA and coefficient-based GCA, the significance of GC values could be calculated.

The permutation test analysis could be used for the significance of GC values. The null distribution of GC values was conducted by random realigning the orders of participants. Then, the normal distribution function was used to calculate the significance of the GC values.

Similar to the SCN, the CaSCN mode supported the analysis of the voxel-/vertex-wise seed-to-brain analysis of volume-/surface-based morphological images and ROI-wise analysis of volume-based morphological images.

Please note that since the large number of the voxel/vertex of morphological images, the permutation analysis of CaSCN in voxel-/vertex-wise seed-to-brain analysis of volume-/surface-based morphological images would cost a related long time and need large memory.

## Modulation Analysis of Structural Covariance Network

Modulation analysis of structural covariance network was based on the GLM. It could evaluate the modulation effect of clinical variables on the structural covariance connectivity. The SurfStat toolkit (see text footnote 4) was applied for the analysis (Lerch et al., 2006; Bernhardt et al., 2009; Worsley et al., 2009).

The formula of the modulation analysis is as follows:

$$Y=\beta_{1}\times X+\beta_{2}\times \text{Var}\,\text{Of}\,\text{Clinic}+\beta_{3}\times X\times \text{Val}\,\text{Of}\,\text{Clinic}Y=\beta_{1}\times X+\beta_{2}\times \text{Var}\,\text{Of}\,\text{Clinic}+\beta_{3}\times X\times \text{Val}\,\text{Of}\,\text{Clinic}$$

$$+\beta_{4}\times \text{Cov}\,\text{Of}\,\text{No}\,\text{Int}+\beta_{4}\times \text{Cov}\,\text{Of}\,\text{No}\,\text{Int}$$

Here, × indicates an interaction between terms, and CovOfNoInt is the covariate of no interest. *Y* is the “Signal” of target ROI, and *X* was the “Signal” of seed ROI. VarOfClinic is the clinical variance for the modulation analysis. The significance of β_*3*_ could represent the power of modulation effect on the structural covariance connectivity between *X* and *Y*.

Similar to the SCN, the MOD-SCN mode supported the analysis of the voxel-/vertex-wise seed-to-brain analysis of volume-/surface-based morphological images and ROI-wise analysis of volume-based morphological images.

## Winner-Take-All and Cortex–Subcotex Covariance Network

Winner-take-all and cortex–subcotex covariance network was introduced in 2020 (Xu et al., 2020). It described the parcelations of the subcortex structure according to the parcelations of the cortex. In the study, the WTA-CSSCN analysis was used to find the differences of cortico-striato-thalamo-cerebellar covariance connectivity between different types of epilepsy patients with generalized tonic–clonic seizures. In the toolkit, the WTA-CSSCN mode included the setup, calculation, group comparison, and result showing.

## Statistical Analysis of Structural Covariance Connectivity

Two kinds of statistical analysis methods were included in the toolkit, the permutation test (Liao et al., 2013; Teipel et al., 2016) and the interaction analysis (Lerch et al., 2006; Xu et al., 2020). These two kinds of methods were only suitable for the comparison of two groups in the toolkit.

In the permutation test, the null distribution was conducted by random disarrangement and regroupment of the two groups of participants. The differences between the regrouped datasets could be used to make up the null distribution, and the significance of the true difference between two groups could be calculated by the normal distribution functions. Let *I*_*A*_ and *I*_*B*_ represent the indices of Group A and Group B, Δ_A−B_ = *I*_A_−*I*_B_ represent the difference between Group A and Group B. Then, Group A and Group B were randomly disarranged and regrouped to be Group A’ and Group B’. $I_{\text{A}}^{{}^{\prime }}$ and $I_{\text{B}}^{{}^{\prime }}$ represent the indices of Group A’ and Group B’, and ${\text{Δ}}_{\text{A}-\text{B}}^{{}^{\prime }}=I_{\text{A}}^{{}^{\prime }}-I_{\text{B}}^{{}^{\prime }}\,$ represent the difference between Group A’ and Group B’. Repeat the random disarrangement and regroupment of Group A and Group B many times, such as 5,000 times. Then, ${\text{Δ}}_{\text{A}-\text{B}}^{{}^{\prime }}$ could build a null distribution. After that, the significance of Δ_A−B_ could be calculated by using the normal distribution function according to the null distribution. The permutation test could be used in the group comparison of SCN, CaSCN, and WTA-CSSCN.

In the interaction analysis, the GLM model was applied in the analysis. It was based on the differences of coefficient of the SCN and MOD-SCN (Bernhardt et al., 2009; Sharda et al., 2016; Xu et al., 2020).

The formula of the SCN group comparison using the interaction analysis is as follows:

$$Y=\beta_{1}\times {\text{Group}}_{1}+\beta_{2}\times {\text{Group}}_{2}+\beta_{3}\times X+\beta_{4}\times {\text{Group}}_{1}Y=\beta_{1}\times {\text{Group}}_{1}+\beta_{2}\times {\text{Group}}_{2}+\beta_{3}\times X+\beta_{4}\times {\text{Group}}_{1}$$

$$\times X+\beta_{5}\times {\text{Group}}_{2}\times X+\beta_{6}\times \text{Var}\,\text{Of}\,\text{Clinic}\times X+\beta_{5}\times {\text{Group}}_{2}\times X+\beta_{6}\times \text{Var}\,\text{Of}\,\text{Clinic}$$

Here,× indicates an interaction between terms, and Cov Of No Int is the covariate of no interest. *X* is the “signal” of the seed region, *Y* is the “signals” of target region, Group1 and Group2 represent the labels of two groups. The difference of β_*3*_ and β_*4*_ presents the difference of covariance connectivity between seed region and target region.

The formula of MOD-SCN group comparison using the interaction analysis is as follows:

$$Y=\beta_{1}\times \text{Group}+\beta_{2}\times X+\beta_{3}\times \text{Var}\,\text{Of}\,\text{Clinic}+\beta_{4}\times \text{Group}Y=\beta_{1}\times \text{Group}+\beta_{2}\times X+\beta_{3}\times \text{Var}\,\text{Of}\,\text{Clinic}+\beta_{4}\times \text{Group}$$

$$\times \text{Val}\,\text{Of}\,\text{Clinic}+\beta_{5}\times X\times \text{Val}\,\text{Of}\,\text{Clinic}+\beta_{6}\times \text{Group}\times X\times \text{Val}\,\text{Of}\,\text{Clinic}+\beta_{5}\times X\times \text{Val}\,\text{Of}\,\text{Clinic}+\beta_{6}\times \text{Group}\times X$$

$$+\beta_{7}\times \text{Group}\times X\times \text{Val}\,\text{Of}\,\text{Clinic}+\beta_{8}\times \text{Cov}\,\text{Of}\,\text{No}\,\text{Int}+\beta_{7}\times \text{Group}\times X\times \text{Val}\,\text{Of}\,\text{Clinic}+\beta_{8}\times \text{Cov}\,\text{Of}\,\text{No}\,\text{Int}$$

Here, × indicates an interaction between terms, and Cov Of No Int is the covariate of no interest. *Y* is the “Signal” of target ROI, and *X* is the “Signal” of seed ROI. Group is the labels of two groups of participants. Val Of Clinic is the clinical variance for the modulation analysis. The significance of β_*7*_ could represent the power of modulation effect on the structural covariance connectivity between *X* and *Y* between two groups of participants.

The interaction analysis in the toolkit was based on the SurfStat Toolkit (see text footnote 4) (Lerch et al., 2006; Bernhardt et al., 2009; Worsley et al., 2009).

## Results-Showing Mode

Six modes of result showing were included in the toolkit: Map (SCN&Modulate), Matrix (SCN&Modulate), Map (GCA), Matrix (GCA), Winner-Take-All, and View for Surf. The Map (SCN&Modulate) and Map (GCA) were suitable for the result showing of SCN, MOD-SCN, and CaSCN of voxel-wise analysis of volume-based morphological images. The Matrix (SCN&Modulate) and Matrix (GCA) were suitable for the result showing of SCN, MOD-SCN, and CaSCN of ROI-wise analysis of the morphological images. The Winner-Take-All was designed for the result showing of WTA-CSSCN. And the View for Surf was suitable for the result showing of the SCN, MOD-SCN, and CaSCN of vertex-wise analysis of surface-based morphological images. For each mode, the parameter results and group comparison results could be shown. In parameter result showing, the related *p* information should be selected for the threshold selection. The permutation *p* result could be shown in each mode independently. The Winner-Take-All mode provided the radar map and cross-sectional slice maps of parcelation and the group comparison result of the permutation test. More detailed description of the result showing would be listed in the help document.

## Demo Dataset

For demo, 52 healthy participants (28 female, age range 20–40 years, 25.82 ± 6.59 years; 24 male, age range 21–46 years, 27.5 ± 7.09 years) were included in the study. All healthy participants were collected in Jinling Hospital, Medical School of Nanjing University. This study was approved by the Medical Ethics Committee in Jinling Hospital, Medical School of Nanjing University. Written informed consent was obtained from all the participants. There was no significant difference in age between the two groups (*T* = −0.884, *p* = 0.381).

All participants were scanned in 3T MRI scanner (Siemens Trio, Germany). High-resolution T1-weighted anatomical images were acquired in the sagittal orientation using a magnetization-prepared rapid gradient-echo sequence with the following parameters: repetition time (TR) = 2,300 ms, echo time (TE) = 2.98 ms, flip angle = 9°, field of view (FOV) = 25.6 × 25.6 cm^2^, acquisition matrix = 256 × 256, slice thickness = 1 mm, 176 slices without interslice gap.

Before being used for the toolkit, the two indices were processed in advance. The GMVs were conducted by CAT12 (see text footnote 6) implemented in SPM12 (see text footnote 2). And the cortical thickness indices were conducted by FreeSurfer 5.3 (see text footnote 3). The TIV was used as the variable of no interest during the following brain structural covariance connectivity analysis.

For a whole view of the software, the showing results of different combinations were listed in Table 1. The sex factor was used as the group label in the group comparison.

**TABLE 1:** The parameters of the demo dataset for BCCT.

Seed	Seed	Template
MNI coordinate 0–53 30, radius 10 mm	MNI coordinate 0–53 30	Automated anatomical labeling
SCN: GMV	SCN: thickness	SCN: GMV
seed-to-brain	seed-to-brain	ROI-wise
Interaction Analysis for Group Comparison	Interaction Analysis for Group Comparison	Permutation Test for Group Comparison
CaSCN: GMV	CaSCN: thickness	CaSCN: GMV
Seed-to-brain, sorted by age	Seed-to-brain, sorted by age	ROI-wise, sorted by age
Residual-based	Coefficient-based	Coefficient-based
Permutation test for group comparison	Permutation test for group comparison	Permutation test for group comparison
Modulation: GMV	Modulation: thickness	Modulation: GMV
Seed-to-brain	Seed-to-brain	ROI-wise
Clinical variable: age	Clinical variable: age	Clinical variable: age
Interaction Analysis for Group Comparison	Interaction Analysis for Group Comparison	Interaction Analysis for Group Comparison

*BCCT, brain covariance connectivity toolkit; CaSCN, causal analysis of structural covariance network; GMV, gray matter volume; ROI, region of interest; SCN, structural covariance network.*

For WTA-CSSCN analysis, we employed a cortical parcelation by dividing bilateral hemispheres into five non-overlapping lobes: (1) frontal lobe, (2) motor/premotor lobe, (3) somatosensory lobe, (4) parietal/occipital lobe, and (5) temporal lobe (Zhang et al., 2008; Ji et al., 2015). The cerebellum from automated anatomical labeling (AAL) (Tzourio-Mazoyer et al., 2002) was set to be the subcortex structure for analysis. In the toolkit, there were templates of thalamus, striatum, and cerebellum available for the WTA-CSSCN analysis. Here, we took the cerebellum for the demo. Additionally, the subcortex did not fully fit the usage range of this mode, since this mode could be used to analyze the winner-take-all and SCN between parcelations of brain part A and regions of brain part B, such as cortex parcelations to the thalamus, parcelations of the thalamus to the cerebellum, etc. The permutation test was used for the comparison of numbers of connected voxels. The sex factor was used as the group label in the group comparison.

## Results of Demo Dataset

Here, we only presented the results of the software. We would not discuss the meaning of the results. The results were presented according to the setting in Table 1.

**FIGURE 3:**
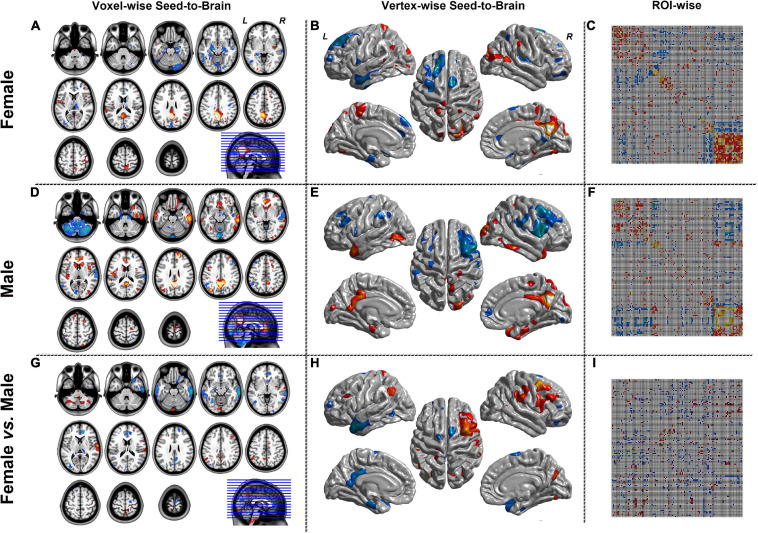
The results of SCN mode. Panels **(A,D,G)** were the results of seed-to-brain connectivity of the GMV. Panels **(B**,**E**,**H)** were the results of seed-to-brain connectivity of thickness. Panels **(C**,**F**,**I)** were the results of ROI-wise connectivity of the GMV with the automated anatomical labeling (AAL) template. All results were set at the threshold of *p* < 0.05 uncorrected.

Figure 3 represented the results of the SCN mode. The left column was the seed-to-brain connectivity maps of volume-based morphological images in the female group, the male group separately, and the group comparison result. The middle column was the seed-to-brain connectivity maps of surface-based morphological images in the female group, the male group separately, and the group comparison result. The right column was the ROI-wise connectivity matrices of volume-based morphological images in the female group, the male group separately, and the group comparison result. All results were set to be *p* < 0.05 uncorrected. The hot color represented the positive values, and the winter color represented the negative values.

**FIGURE 4:**
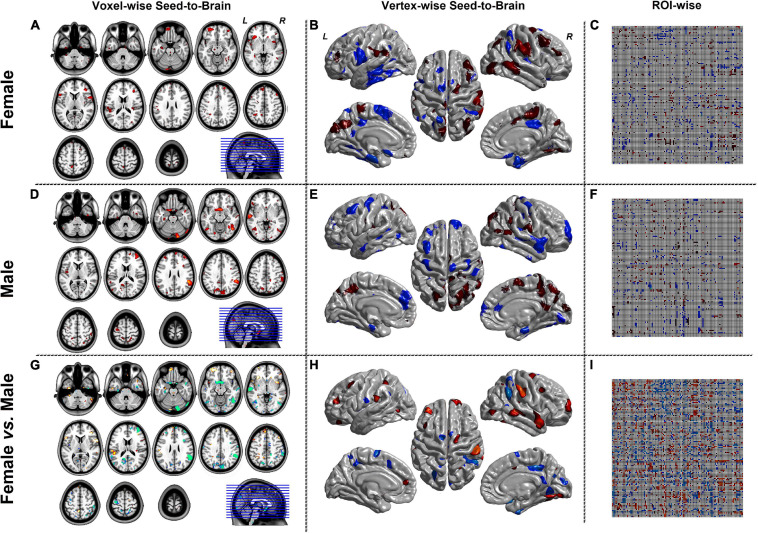
The results of CaSCN mode with X (seed)-to-Y (Brain/Target ROI). Panels **(A,D,G)** were the results of seed-to-brain connectivity of the GMV. Panels **(B**,**E**,**H)** were the results of seed-to-brain connectivity of thickness. Panels **(C**,**F**,**I)** were the results of ROI-wise connectivity of the GMV with the AAL template. All results were set at the threshold of *p* < 0.05 uncorrected.

**FIGURE 5:**
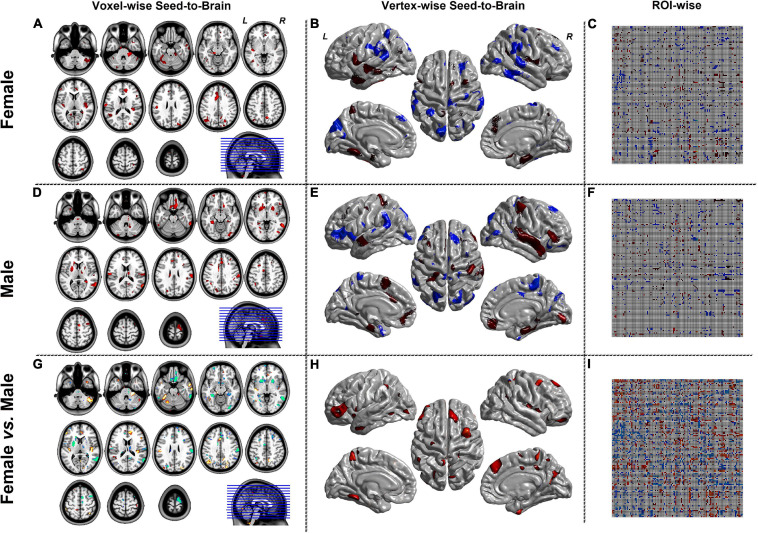
The results of CaSCN mode with Y (Brain/Target ROI)-to-X (seed). Panels **(A**,**D**,**G)** were the results of seed-to-brain connectivity of the GMV. Panels **(B**,**E**,**H)** were the results of seed-to-brain connectivity of thickness. Panels **(C**,**F**,**I)** were the results of ROI-wise connectivity of the GMV with the AAL template. All results were set at the threshold of *p* < 0.05 uncorrected.

Figures 4, 5 were the results of the CaSCN mode. Figure 4 represented the out effect of GCA of the seed region, and Figure 5 represented the in effect of GCA of the seed region. Similar to the SCN result-showing mode (Figure 3), the left column was the results of seed-to-brain connectivity maps of volume-based morphological images, the middle column was the results of seed-to-brain connectivity maps of surface-based morphological images, and the right column was the results of ROI-wise connectivity matrices of volume-based morphological images. All results were presented as the female group (top), the male group (middle), and the comparison result (bottom). All results were set to be *p* < 0.05 uncorrected. The hot color represented the positive values, and the winter color represented the negative values.

**FIGURE 6:**
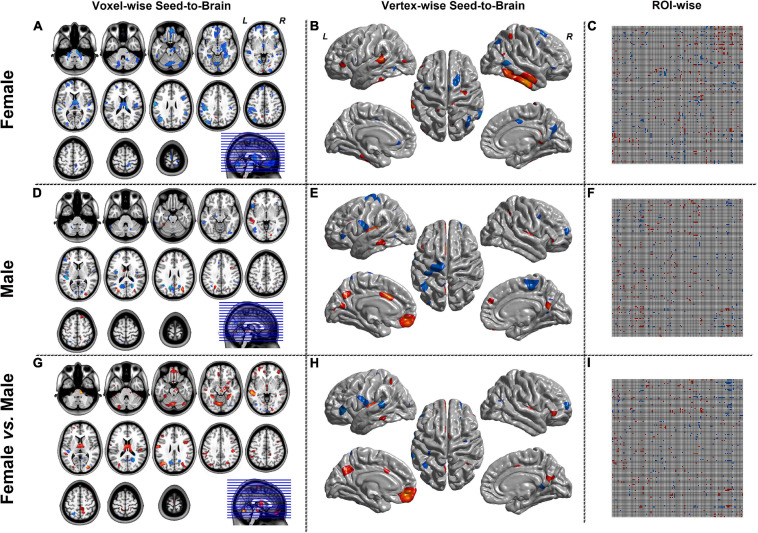
The results of modulation mode with X (seed)-to-Y (Brain/Target ROI). Panels **(A**,**D**,**G)** were the results of seed-to-brain connectivity of the GMV. Panels **(B**,**E**,**H)** were the results of seed-to-brain connectivity of thickness. Panels **(C**,**F**,**I)** were the results of ROI-wise connectivity of the GMV with the AAL template. All results were set at the threshold of *p* < 0.05 uncorrected. The ROI-wise connectivity contained the seed-to-Target and Target-to-seed effects.

Figure 6 represented the results of the MOD-SCN mode. Similar to the SCN result-showing mode (Figure 3), the left column was the results of seed-to-brain connectivity maps of volume-based morphological images, the middle column was the results of seed-to-brain connectivity maps of surface-based morphological images, and the right column was the results of ROI-wise connectivity matrices of volume-based morphological images. All results were presented as the female group (top), the male group (middle), and the comparison result (bottom). All results were set to be *p* < 0.05 uncorrected. The hot color represented the positive values, and the winter color represented the negative values. The seed-to-brain results represented the modulation effect of the connectivity with seed region to whole brain. The upper triangular of the ROI-wise connectivity matrix represented the seed-to-target effects, while the lower triangular represented the target-to-seed effects. It was an asymmetric matrix.

**FIGURE 7:**
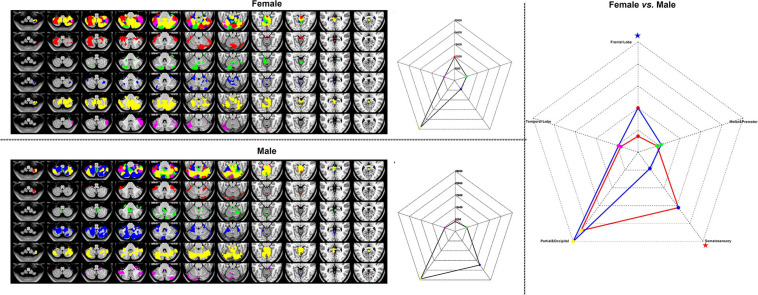
The results of WTA-CSSCN mode. Red, frontal lobe; green, motor and premotor; blue, somatosensory; yellow, parietal and occipital; and violet, temporal lobe. The red star represented the female group > male group, and the blue star represented the male group < female group.

Figure 7 represented the results of the WTA-SCN mode with the female group (upper left), the male group (left bottom) separately, and the group comparison result (right). The colors in the cerebellum represented the corresponding brain regions of the cortex. The group comparison represented the significance of the numbers of cortex ROI-to-cerebellum connectivity between female and male groups.

## Discussion

In the current work, we developed a user-friendly GUI BCCT in MATLAB platform. We also present the results of BCCT toolkit with the demo dataset. A completed workflow of structural covariance connectivity analysis was presented to the researchers who would like to use the toolkit.

As far as we know, this toolkit is the first software dedicated to structural covariance analysis. Additionally, for the first time, the toolkit integrated the group comparison, which formerly restricted the application of structural covariance analysis for clinical researchers. Furthermore, the advanced methods, CaSCN, MOD-SCN, and WTA-CSSCN, have extended the application areas of SCN. Structural covariance analysis holds great promise in many areas of neuroscience (Bermudez et al., 2009; Lv et al., 2010; Zielinski et al., 2010) and neuropsychiatric disorders (Ge et al., 2020; Zhang et al., 2020).

The result showings of SCN, CaSCN, and MOD-SCN of demo dataset could help researchers to be familiar with the toolkit. Furthermore, the volume-based and surface-based analysis could help meet more requirements of the researcher. The WTA-CSSCN could be used for the parcelation of subcortex structures (Xu et al., 2020). The MOD-SCN connected the covariance connectivity and the clinical variables (Bernhardt et al., 2009; Xu et al., 2020). The statistical analysis of structural covariance connectivity could give the researcher more useful information.

The results of the toolkit could be used for the later analysis of other toolkits, such as the graph theoretic analysis. Additionally, the functional covariance connectivity, such as hemodynamic (Tzourio-Mazoyer et al., 2002), metabolic (Di and Biswal, 2012), and amplitude of low-frequency fluctuation (Zhang et al., 2011b; Taylor et al., 2012; Liao et al., 2013) descriptor, could be done in the toolkit. The text-based and mat-based mode of ROI-wise connectivity could be used for other kinds of neuroimage mode, such as electroencephalogram (EEG) signals of sensors.

## Methodological Consideration

For the limitation of time and abilities of the developer, there were several methodological considerations in the toolkit. Firstly, the surface-based analysis was only limited to the data structures of FreeSurfer software. The support of other types of surface-based images would be added in future versions. Secondly, the group comparison was restricted into two groups, and only two kinds of statistical analysis tools were included in the toolkit. The statistical analysis beyond two groups and other statistical tools need to be discussed later. Additionally, some other kinds of covariance connectivity analysis and statistical tools require new ideas from the user of the toolkit. The result-showing mode of the toolkit needed further update in the later version.

## Data Availability Statement

The raw data supporting the conclusions of this article will be made available by the authors, without undue reservation.

## Ethics Statement

The studies involving human participants were reviewed and approved by Jinling Hospital, School of Medicine, Nanjing University. The patients/participants provided their written informed consent to participate in this study.

## Author Contributions

QX and QZ: draft writing. GLi, X-jD, XX, JH, QY, RL, ZiZ, and YY: data collection. GLi and X-jD: data process. ZhZ and GLu: topic propose. RQ and LZ: draft modification. QX, QZ, and XD: software developer. All authors contributed to the article and approved the submitted version.

## Conflict of Interest

The authors declare that the research was conducted in the absence of any commercial or financial relationships that could be construed as a potential conflict of interest.

## Footnotes

1 https://github.com/JLhos-fmri/BrainCovarianceConnectToolkitV2.1

2 https://www.fil.ion.ucl.ac.uk/spm/

3 https://www.freesurfer.net/

4 http://www.math.mcgill.ca/keith/surfstat/#ICBMagain

5 http://dbm.neuro.uni-jena.de/wordpress/vbm/

6 http://www.neuro.uni-jena.de/cat/
